# Physical profiling in elite professional men’s football: Academy, reserve and first team variations in fitness and performance

**DOI:** 10.1080/11791543.2026.2697533

**Published:** 2026-07-04

**Authors:** Thomas Carpels, Ole J. Kemi

**Affiliations:** a School of Cardiovascular and Metabolic Health, College of Medical, Veterinary and Life Sciences, University of Glasgow, Glasgow, UK; b Royal Antwerp Football Club, Performance Department, Antwerp, Belgium

**Keywords:** Football, soccer, academy, reserve team, first team, athlete profile

## Abstract

We profiled the physical fitness and training and match performance of elite academy (under-18 (U18, *n* = 23), reserve (RES, *n* = 15)), and first teams (FT, *n* = 25) to assess physical readiness for academy-FT progression. Further, we segregated players into player positions, ages, and current versus former academy players that had successfully progressed to FT. Aerobic capacity, anthropometry, and global positioning system- and heart rate-derived performance profiles in training and matches quantified external and internal loads. FT demonstrated 10% more body mass (*p* < 0.01) with similar adiposity, and ~10% higher aerobic capacity (*p* < 0.01) versus academy. In training, U18 covered more distances: 727 m (16%) total distance, 115 m (48%) high-speed, and 65 m (79%) sprint distance, and generated 0.63  m/s (8%) higher peak speeds versus FT (all *p* < 0.01), while FT displayed higher mean speeds: 8 m/min (11%) and 18 m/min (24%) versus U18 and RES (all *p* < 0.05). In terms of matches, U18 and RES covered more distance: total 539 m (5%) and 567 m (6%) and high-speed distance: 111 m (18%) and 121 m (20%), and displayed higher mean speeds: 13 m/min (11%) and 14 m/min (12%), versus FT (all *p* <  0.05). Thus, in this club, FT players demonstrated higher aerobic capacity, but not physical performance in training or matchplay. Academy players were not inferior, but displayed different performance behaviours.

## Introduction

Football places extensive physical demands on players, making physical fitness a key determinant of performance (Bangsbo et al., 2006; Sporis et al., 2009; Waldron & Murphy, 2013). Higher levels of physical fitness are associated with higher matchplay performance (Koudellis et al., 2024), team success (Apor, 1988), and increased capacity to sustain high intensity in training and match, regardless of development and competition level (Barnes et al., 2014; Bradley et al., 2009; Bush et al., 2015; Carpels et al., 2021; Dugdale et al., 2021; Helgerud et al., 2001; Houtmeyers et al., 2021). The focus on physical fitness has further become increasingly important because of fixture-congestions and elevated match demands following increased intensity of play (Clemente et al., 2021; Dellal et al., 2015; Malone et al., 2015; Nassis et al., 2020), including in academy football (Burgess et al., 2012).

Despite a structured development and progression pathway from academy to reserve to first team, only a small proportion of players achieve this (Carpels et al., 2021; Dugdale et al., 2021). A major cause of this is systemic and individual constraints. At the systemic level, discrepancies in training and match demands between academy, reserve, and first teams lead to different microcycles, even within the same club, which result in mismatched load profiles that may inadequately prepare players for first team transition (Carpels et al., 2024; Swallow et al., 2021). At the individual level, players may lack the anthropometric characteristics, physical capacity, or performance outputs required at the first team level, including the ability to consistently perform high-intensity actions and sustain high-intensity workloads (Milsom et al., 2015; Veale et al., 2010), especially in selected player positions where specific physical demands may dictate player selection (Bloomfield et al., 2007; Bush et al., 2015; Di Salvo et al., 2007; Martin-Garcia et al., 2018).

These differences highlight the importance of physical profiling. Characterization of anthropometric and external and internal load parameters provides a robust framework for identifying developmental gaps and benchmarking progression toward elite performance standards (Houtmeyers et al., 2021). While previous studies have examined differences across competition levels and training contexts (Carpels et al., 2024; Koudellis et al., 2024; Martin-Garcia et al., 2018; Swallow et al., 2021), no study has yet systematically compared these physical profiles across academy, reserve and first teams within the same professional elite environment, including after segregation for player positions, age groups and players that did or did not achieve success.

Player development and progression are multifactorial (Morgans et al., 2023), but the above indicates a substantial physical dimension. We propose that comparing physical player profiles across the different cohorts establishes benchmarks for physical standards that academy and reserve players must meet in order to compete at the higher level (Cone, 2012; Sporis et al., 2009). The aim of this study was therefore to compare the anthropometric characteristics, physical fitness, and external and internal load parameters between academy, reserve and first-team players within the same elite professional environment. Analyses were further stratified by player positions, age groups, and success in transitioning to the first team by comparing current academy and reserve players to players who had previously achieved this progression.

## Materials and methods

### Participants

**Figure 1.**
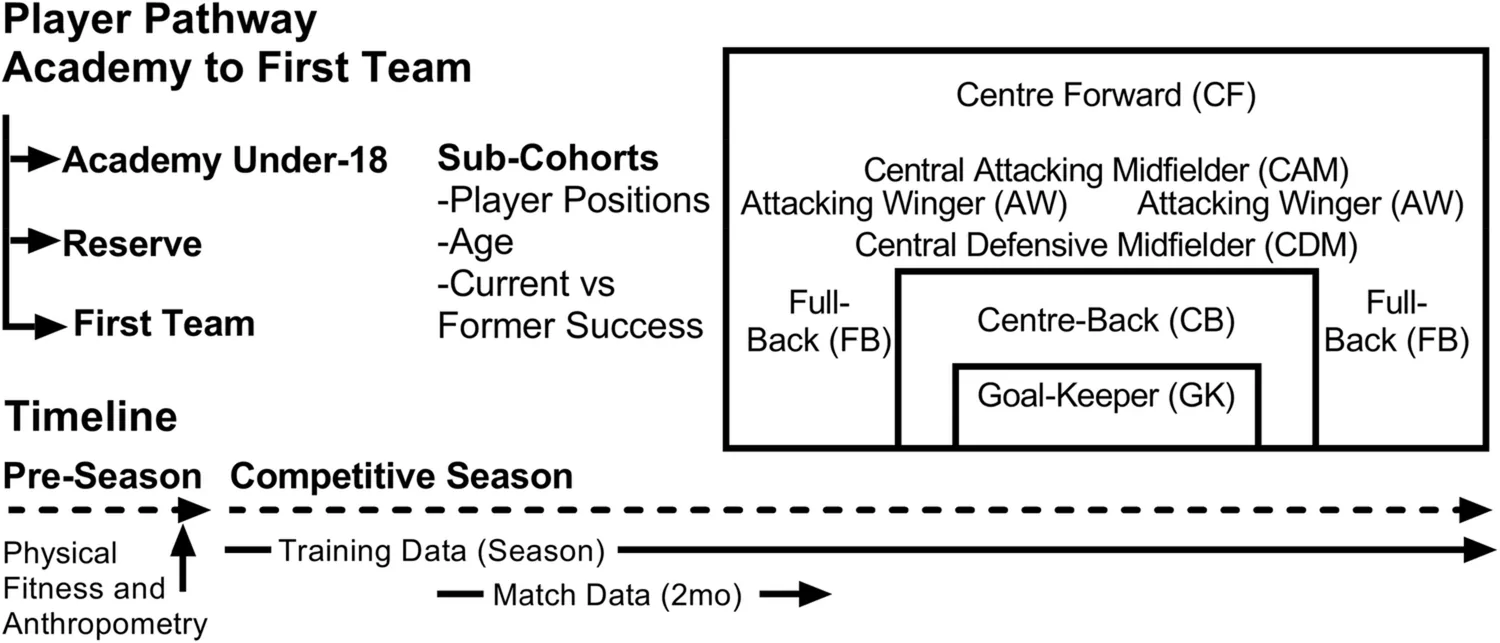
Study design with teams and subgroup analyses including player positions, and timings of data capture periods. Abbreviations: mo = months.

Full-time professional elite academy consisting of under-18 and reserve teams and senior first team football players in a male Scottish Premier League club during the 2018–2019 season were included, covering all on-field playing positions, with player positions, player numbers and characteristics shown in Figure 1 and Table 1. Player assignment was decided by the club. All academy players had a minimum of 1-year employment history within the club and therefore were following club health screening, physical conditioning and training protocols. All teams competed in the premier domestic (Scottish Football Association League and Cup) and international competitions (UEFA Europa League). The study conformed to the Declaration of Helsinki, all procedures were approved by the Institutional Review Board (College of Medical, Veterinary and Life Sciences Ethics Committee, University of Glasgow, FBLS1006), and all players, including parents/guardians for under-18 players, provided written informed consent.

**Table 1.** Player characteristics of elite academy under-18 (U18) and reserve (RES) teams and first team (FT).

	GK			FB			CB			CDM			CAM			AW			CF			TEAM		
Number of players	U18 (3)	RES (2)	FT (3)	U18 (5)	RES (3)	FT (5)	U18 (4)	RES (4)	FT (4)	U18 (3)	RES (2)	FT (4)	U18 (3)	RES (1)	FT (2)	U18 (3)	RES (2)	FT (5)	U18 (2)	RES (1)	FT (2)	U18 (23)	RES (15)	FT (25)
Age (years)	16.7 ± 0.6*#	19.0 ± 0.0*	26.3 ± 4.0	16.6 ± 0.5*#	18.0 ± 0.0*	23.8 ± 4.7	16.0 ± 1.4*#	18.5 ± 0.6*	28.8 ± 8.7	16.0 ± 1.0*#	18.0 ± 0.0*	24.0 ± 3.8	16.7 ± 0.6*	18.0/	26.5 ± 4.9	16.7 ± 0.6*#	19.0 ± 0.0*	27.2 ± 5.3	16.0 ± 0.0*	19.0/	26.5 ± 6.4	16.4 ± 0.8**##	18.5 ± 0.5**	26.0 ± 5.2
Height (cm)	190.2 ± 9.2*	186.7 ± 0.6*	177.8 ± 4.7	176.3 ± 5.2	178.0 ± 5.3	186.2 ± 5.8	182.5 ± 3.0	188.6 ± 2.7	182.4 ± 7.2	178.7 ± 3.8	182.1 ± 2.6	184.2 ± 6.3	174.8 ± 2.3	175.8/	180.1 ± 4.0	167.7 ± 2.0*	169.7 ± 0.8*	184.6 ± 5.7	175.3 ± 2.9	179.7/	186.8 ± 9.0	178.1 ± 7.6*	181.4 ± 4.0	183.4 ± 6.0
Body Mass (kg)	75.6 ± 9.8	79.9 ± 4.7	72.2 ± 4.6	64.6 ± 5.1*	70.4 ± 4.9*	81.3 ± 5.5	73.2 ± 5.6#	82.0 ± 4.1	79.0 ± 4.3	66.5 ± 5.5	74.4 ± 3.8	76.0 ± 8.1	68.7 ± 3.0	66.4/	78.3 ± 1.0	64.7 ± 7.5*	62.5 ± 7.8*	81.6 ± 5.5	71.8 ± 8.6	78.0/	84.1 ± 5.2	69.0 ± 6.9**	74.5 ± 8.0*	79.0 ± 5.9
Skinfolds (mm)	59.8 ± 16.6	78.7 ± 11.5	48.6 ± 3.6	47.7 ± 4.8	53.3 ± 13.5	52.3 ± 5.8	58.3 ± 2.7	61.5 ± 10.9	50.0 ± 10.4	60.6 ± 12.7	42.8 ± 5.9	60.0 ± 17.9	56.4 ± 12.8	62.6/	42.9 ± 0.4	45.5 ± 5.3	46.6 ± 0.9	51.5 ± 22.2	70.2 ± 3.0	49.6/	47.9 ± 5.9	55.6 ± 10.8	56.9 ± 13.8	51.4 ± 12.8
vIFT (km/h)	20.0 ± 0.0	19.5/	18.8 ± 1.3	21.5 ± 0.5	21.5 ± 0.0	21.3 ± 0.6	20.3 ± 1.2	20.5 ± 0.5	21.0 ± 1.4	20.8 ± 0.8	20.8 ± 0.4	21.8 ± 0.4	21.0/	//	22.0/	21.2 ± 0.8	14.0/	20.8 ± 1.0	20.3 ± 0.4	//	18.0/	20.8 ± 0.8	19.9 ± 2.3	20.6 ± 1.5
V̇O_2max_ (mL/kg/min)	51.2 ± 0.2	50.9/	52.3 ± 0.8	54.6 ± 1.2*	55.0 ± 0.1*	58.5 ± 1.4	51.6 ± 1.2	52.7 ± 0.8	56.1 ± 2.4	52.9 ± 1.7	53.4 ± 0.6	58.2 ± 3.1	53.8/	//	62.6/	53.9 ± 1.5	40.0/	57.0 ± 1.1	51.6 ± 0.4	//	48.5/	53.0 ± 1.9**	51.8 ± 4.6**	56.3 ± 3.6
V̇O_2max_ (mL/kg^0.75^/min)	150.7 ± 6.4	153.7/	156.5 ± 1.2	154.8 ± 5.0*	160.9 ± 0.7*	173.5 ± 4.0	150.9 ± 10.2	159.5 ± 2.9	170.9 ± 5.0	151.1 ± 6.5	156.9 ± 3.8	171.4 ± 6.7	152.9/	//	184.4/	152.8 ± 4.2	110.0/	167.7 ± 1.4	150.0 ± 5.6	//	147.6/	152.2 ± 5.8**	153.1 ± 16.4**	167.7 ± 9.5
HR_max_ (bpm)	//	193 ± 1	//	200 ± 3	197 ± 5	188 ± 11	200 ± 3	197 ± 5	193 ± 12	203 ± 10	201 ± 13	202 ± 8	196 ± 3	204/	197 ± 8	195/	203 ± 9	194 ± 9	203 ± 4	194/	180 ± 4	200 ± 5*	198 ± 7*	193 ± 10

Notes: The table shows the physical characteristics of players on different teams, including those after segregation to player positions. Abbreviations: U18 = under-18 team, RES = reserve team, FT = first team, GK = goalkeeper, FB = full back, CB = centre back, CDM = central defensive midfielder, CAM = central attacking midfielder, AW = attacking winger, CF = centre forward, vIFT = maximal speed at 30:15 intermittent fitness test, V̇O_2max_ = maximal oxygen uptake, HR_max_ = maximal heart rate, bpm = beats per minute. Statistical notes: Data are presented as the means ± standard deviation. Statistical difference between teams (U-18 versus RES versus FT) after Benjamini‒Hochberg correction: * = *p*<0.05, ** = *p*<0.01 versus FT; # = *p*<0.05, ## = *p*<0.01 versus RES.

### Study design

The study included cross-sectional comparisons using data collected prospectively across one season, with longitudinal capture of training and match performances. Training data were recorded throughout the competitive season, while match data were recorded during a 2-month competitive period where academy, reserve and first-team fixtures and results were comparable and thus had equal opposition effects and result influences. Anthropometry and physical fitness were measured at the end of pre-season due to the availability of simultaneous testing across teams and because physical performance tends to peak at or around this time (Meckel et al., 2018) (Figure 1). Deconditioned or injured players were excluded, while GK data were included for player characteristics, but excluded from training and match physical performances because of different training and match requirements. Individual outfield player sessions were also excluded where club management dictated a departure from a normal training schedule. The number of observations is provided in Tables 2 and 3. Prior to each test, training session or match, players warmed-up following the club protocol.

**Table 2.** Physical performance during training in elite academy under-18 (U18) and reserve (RES) teams and first team (FT).

	FB			CB			CDM			CAM			AW			CF			TEAM		
	U18	RES	FT	U18	RES	FT	U18	RES	FT	U18	RES	FT	U18	RES	FT	U18	RES	FT	U18	RES	FT
Number of observations	*(26)*	*(13)*	*(36)*	*(19)*	*(16)*	*(32)*	*(11)*	*(6)*	*(30)*	*(26)*	*(7)*	*(15)*	*(7)*	*(9)*	*(27)*	*(16)*	*(2)*	*(19)*	*(105)*	*(53)*	*(159)*
Total distance (m)	3027 ± 697##	4335 ± 669**	3346 ± 1068	5313 ± 824*#	4339 ± 1154	4441 ± 1144	5039 ± 694**#	3675 ± 733	2748 ± 1112	5854 ± 1278	5255 ± 706	4766 ± 1026	6644 ± 616##	4794 ± 1465	5758 ± 1038	3737 ± 1388	3118 ± 423	3155 ± 1307	4701 ± 1576**	4415 ± 1087	3974 ± 1518
High-speed distance (5.5–7 m/s, m)	51 ± 44	104 ± 160	92 ± 106	339 ± 178**##	152 ± 102	153 ± 114	291 ± 169**	155 ± 292	72 ± 67	322 ± 167**	213 ± 67	168 ± 103	510 ± 154**##	145 ± 56	179 ± 74	134 ± 226	42 ± 9	102 ± 153	239 ± 209**##	143 ± 140	124 ± 110
Sprint Distance (>7 m/s, m)	7 ± 13	6 ± 9	15 ± 29	131 ± 160**	31 ± 53	31 ± 32	64 ± 133*	7 ± 16	1 ± 4	129 ± 99**##	14 ± 38	31 ± 37	134 ± 104**	71 ± 67	22 ± 24	55 ± 116	0/	5 ± 15	82 ± 118**##	25 ± 48	17 ± 28
Peak speed (m/s)	6.75 ± 0.73	6.95 ± 0.57	5.80 ± 1.99	7.68 ± 0.66	7.20 ± 0.94	7.43 ± 0.63	7.36 ± 0.63*#	6.62 ± 0.61	6.59 ± 0.49	7.95 ± 0.65#	6.80 ± 0.64	7.50 ± 0.61	7.92 ± 0.62	7.93 ± 1.30	7.55 ± 0.59	7.51 ± 1.77	6.08 ± 0.09	6.71 ± 0.66	7.47 ± 1.01**	7.10 ± 0.94	6.84 ± 1.26
Mean speed (m/min)	58 ± 7**	64 ± 16*	89 ± 23	69 ± 14#	51 ± 12**	75 ± 11	66 ± 11##	47 ± 7	61 ± 11	73 ± 10#	59 ± 10*	75 ± 7	78 ± 10	67 ± 8*	82 ± 10	66 ± 13#	43 ± 6	63 ± 11	67 ± 12*#	57 ± 14*	75 ± 17
Time > 85% HR_max_ (min:sec)	3:59 ± 4:19*	10:09 ± 8:50	9:28 ± 10:57	9:43 ± 6:54	6:50 ± 9:21	9:03 ± 6:27	10:19 ± 5:28	4:36 ± 4:48	7:33 ± 8:49	14:18 ± 10:45 #	4:16 ± 6:30	11:05 ± 7:27	18:26 ± 6:08	14:51 ± 11:02	18:38 ± 10:20	8:23 ± 10:15	5:10 ± 2:49	9:04 ± 10:00	9:53 ± 8:55	8:21 ± 9:04	10:41 ± 9:50

Notes: Table shows physical performance outputs for external and internal loads during training for players of different teams, including after segregation to player positions, excluding goalkeepers. Abbreviations: U18 = under-18 team, RES = reserve team, FT = first team, FB = full back, CB = centre back, CDM = central defensive midfielder, CAM = central attacking midfielder, AW = attacking winger, CF = centre forward, HR_max_ = maximal heart rate. Statistical notes: Data are presented as the means ± standard deviation. Statistical difference between teams (U-18 versus RES versus FT) after Benjamini‒Hochberg correction: * = *p*<0.05, ** = *p*<0.01 versus FT; # = *p*<0.05, ## = *p*<0.01 versus RES.

**Table 3.** Physical performance during a match in elite academy under-18 (U18) and reserve (RES) teams and first team (FT).

Number of observations	FB			CB			CDM			CAM			AW			CF			TEAM		
	U18 *(6)*	RES *(7)*	FT *(15)*	U18 *(5)*	RES *(2)*	FT *(15)*	U18 *(4)*	RES *(4)*	FT *(8)*	U18 *(4)*	RES *(7)*	FT *(13)*	U18 *(4)*	RES *(8)*	FT *(15)*	U18 *(4)*	RES *(5)*	FT *(7)*	U18 *(27)*	RES *(33)*	FT *(73)*
Total distance (m)	10711 ± 773	10974 ± 877	10179 ± 592	9851 ± 710	10449 ± 167	9317 ± 876	11140 ± 409	11210 ± 372	11261 ± 462	10381 ± 1268	10969 ± 1392	10956 ± 750	10778 ± 976	10641 ± 909	10525 ± 660	10444 ± 1019	10579 ± 942	8778 ± 1533	10734 ± 1007*	10762 ± 987*	10195 ± 1105
High-speed Distance (5.5–7m/s, m)	751 ± 137**	718 ± 154**	516 ± 125	395 ± 121	446 ± 144	357 ± 94	552 ± 151	537 ± 132	421 ± 95	592 ± 137	603 ± 121	599 ± 159	699 ± 117	708 ± 117	603 ± 110	671 ± 82	658 ± 80	468 ± 193	612 ± 155**	622 ± 159**	501 ± 156
Sprint distance (>7 m/s, m)	245 ± 86	208 ± 88	162 ± 52	85 ± 33	83 ± 50	72 ± 36	93 ± 55	94 ± 46	66 ± 40	116 ± 63	101 ± 58	140 ± 60	216 ± 90	260 ± 116	249 ± 81	144 ± 90	159 ± 92	180 ± 92	148 ± 91	157 ± 99	149 ± 88
Peak speed (m/s)	8.73 ± 0.59	8.58 ± 0.54	8.71 ± 0.35	8.32 ± 0.54	8.50 ± 0.80	8.32 ± 0.48	8.00 ± 0.31	8.11 ± 0.28	8.19 ± 0.41	8.14 ± 0.42	8.22 ± 0.55	8.35 ± 0.40	8.76 ± 0.64	8.86 ± 0.56	8.82 ± 0.23	8.17 ± 0.51	8.46 ± 0.68	8.60 ± 0.40	8.41 ± 0.58	8.42 ± 0.56	8.51 ± 0.44
Mean speed (m/min)	119 ± 9*	122 ± 10*	106 ± 6	109 ± 8	116 ± 2*	97 ± 9	124 ± 5	125 ± 4	118 ± 5	115 ± 14	122 ± 15	114 ± 9	120 ± 11	118 ± 10	110 ± 7	116 ± 11*	118 ± 10*	91 ± 16	119 ± 11*	120 ± 11*	106 ± 12

Notes: The table shows physical performance outputs for external load during a match in players of different teams, including after segregation to player positions, excluding goalkeepers. Abbreviations: U18 = under-18 team, RES = reserve team, FT = first team, FB = full back, CB = centre back, CDM = central defensive midfielder, CAM = central attacking midfielder, AW = attacking winger, CF = centre forward, HR_max_ = maximal heart rate. Statistical notes: Data are presented as the means ± standard deviation. Statistical difference between teams (U-18 versus RES versus FT) after Benjamini‒Hochberg correction: * = *p*<0.05, ** = *p*<0.01 versus FT.

### Anthropometry and physical fitness

Age and body height and mass were recorded and adiposity was assessed from subcutaneous fat skinfold recordings (Harpenden Skinfold Caliper, HaB Direct, Warwickshire, UK) by averaging a minimum of 2 recordings each of triceps, subscapular, biceps, iliac crest, supraspinale, abdominal, front thigh and medial calf sites by the same experienced observer; as validated in football (Reilly et al., 2009). The calculation of intra-observer technical error of measurements (TEM; Ulijaszek & Kerr, 1999) showed high precision, with a relative TEM ranging 2.1%–5.4%.

The 30:15 Intermittent Fitness Test (IFT) was used to assess football-specific aerobic and anaerobic endurance capacity and 180° change of direction ability. This test has previously demonstrated excellent test–retest reliability for measuring both maximal velocity and maximal heart rate (HR_max_) and thus determining physical fitness in a variety of comparable team sports study groups, with intraclass correlation coefficients (ICC) ranging 0.80–0.99 (with the majority of comparisons ranging 0.90–0.99), and coefficients of variation ranging 1.5%–6.0% (Grgic et al., 2021), and has been found to be valid for estimating cardiorespiratory fitness (Buchheit et al., 2011). Maximal performance was defined when players consecutively failed to reach the required zone 3x, whereupon the velocity of the last completed stage (V_IFT_) was recorded, while maximal oxygen uptake (V̇O_2max_) was estimated as: V̇O_2max_ = 28.3−(2.15xG)−(0.741xA)-(0.0357xW) + (0.0586xAxV_IFT_) + (1.03xvIFT), with G = gender (male: 1), A = age (years), W = body mass (kg), and V_IFT_ = maximal velocity of IFT (km/h) (Buchheit, 2008). V̇O_2max_ was allometrically scaled to body mass (mL/kg^0.75^/min) to appropriately compare players with different body masses (Helgerud et al., 2001). Heart rate (HR) was continuously measured using Polar Team Pro heart rate monitors with chest straps positioned according to the manufacturer’s specifications (Polar Electro, Kempele, Finland), with 1  Hz data logging following 1000  Hz capture, which was previously validated and used in Football (Dalen & Loras, 2019; Gilgen-Ammann et al., 2019). The highest recording was defined as HR_max_.

### Training

On-pitch physical conditioning, match preparation and recovery sessions were analysed, whereas technical/tactical drills and indoor gym-based strength and conditioning sessions were excluded. For most of the season, microcycles consisted of the first team playing 2 matches/week, whereas both academy teams played 1 match/week, which resulted in different load profiles, but which are common (Carpels et al., 2024; Martin-Garcia et al., 2018; Swallow et al., 2021). This also affects training schedules and regimens, with the first team experiencing fewer and/or shorter training sessions versus academy teams. As a result, typical weeks included 3–4 analysed training sessions from the first team and 4 from academy teams, but these were longer than the first team sessions. All the training was conducted on grass pitches and was measured by Catapult OptimEye X4 Athlete Monitoring System (Catapult Sports, Melbourne, Australia) global positioning system (GPS, 10 Hz) and triaxial accelerometry (100 Hz), which were previously reported to be valid and reliable for this purpose, with ICC ranging 0.51–0.99 (with the majority of comparisons in 0.80–0.99 range), a %-age typical error ranging 0.8–4.8, and no difference from higher-frequency sampling (Johnston et al., 2014). To avoid interunit errors, which are typically the major source of error (Theodoropoulos et al., 2020), each player was assigned their own specific device. The mean number of connected satellites during the recordings was 14 ± 1, and the horizontal dilution of precision (HDOP) was 0.73 ± 0.04. Devices were activated ~10 min prior to each session and worn according to the manufacturer’s instructions in purpose-designed vests. Individual rehabilitation or gym sessions, and players involved in sessions outside their team designation were excluded. The measurements included total distance (m), high-speed distance (5.5–7 m/s; m), sprint distance (>7 m/s; m), peak speed (m/s), mean speed (m/min), and time spent >85% of HR_max_ (min:sec). Time spent >85% of HR_max_ was analysed as exercise above this threshold represents a high-intensity cardiovascular load in youth football (Tounsi et al., 2024).

### Match

Matches were analysed from a 2-month period in the first half of the season (mid-October to mid-December), where all teams experienced 2-matches/week microcycles. Sixteen matches from all teams were analysed, including domestic (11 league, 1 cup) and international (5 Europa League) matches, with academy teams following the first team schedule. Measurements replicate those described above, except that the HR was not measured during the matches. Player data with < 60 mins/match were excluded, and player data from >60 to <90 mins/match were normalised to the full length of that specific match.

### Age-dependent effects

We extracted match physical performance data from defined age groups within the different teams. This allowed us to examine age-dependent effects by comparing ≤16 years versus >16 years in under-18 team players, and ≥18 years reserve players versus ≤23 years first team players, with cut-points informed by documented maturational transitions in elite youth football (Tounsi et al., 2024).

### Analysis of successful former academy players

Finally, we compared current to former academy players who progressed to first-team contracts and subsequently featured regularly in first-team matches. The inclusion criteria required the availability of comparable measurements in terms of duration and seasonal timing, which were collected during their academy phase. Players whose development occurred under substantially different academy training or physical profiling frameworks were excluded to ensure contextual consistency. This approach yielded a sample of 4 successfully transitioned players and included data on player characteristics as well as training (24 sessions) and match (34 matches) performances. Contemporary data were collected as described above, but historical data were supplemented with measurements captured with FieldWiz (ASI, Lausanne, Switzerland) and PlayerTek (Catapult Sports, Melbourne, Australia), which were in use at that time. The inclusion of these data was considered necessary to enable a rare and practically valuable longitudinal comparison of current versus former successful academy players within the same club environment.

### Statistical analysis

Training and match data were analysed with the OpenField Console Software (version 1.14, Catapult Sports, Melbourne, Australia), while statistical analysis was conducted using R (version 3.6.1, R Core Team). Sample size determination was deemed impractical since player logistics were managed by club, but sample size calculation for the necessary number of players to identify expected differences between teams, based upon expected minimal team differences and standard deviation (SD) of measurements of total, high-speed and sprint distances, mean speed, V_IFT_ and V̇O_2max_, from our own previous experience (Carpels et al., 2024) and published studies with similar designs (e.g., Houtmeyers et al., 2021), with *α* = 0.05 and *β* = 0.2 (80% power), indicated that each team required 10–12 players. This allowed us to divide the elite academy into under-18 and reserve teams. Furthermore, the sample size calculation for the number of observations/team based upon the same criteria as above suggested ~20 observations/team. The normal distribution of the data was confirmed by the Shapiro‒Wilk test *p* > 0.05 (observed range *p* = 0.11–0.64), and by observing the diagonal data distribution in Q‒Q plots.

Comparisons between 2 cohorts were conducted using 2-tailed unpaired t-tests with unequal variances. Comparisons across 3 cohorts were conducted using linear mixed models with teams (under-18, reserve, first team) and player positions as fixed factors and individual player identity included as a random factor to account for repeated observations within individuals across training sessions and matches. The estimation method was restricted to maximum likelihood, and degrees of freedom were calculated by the Satterthwaite approximation. Where significant main effects occurred, pairwise Sidak post-hoc comparisons were conducted to identify those effects. Current and former academy players were compared by non-parametric Mann–Whitney U tests because of the small sample size. Statistical significance was set at *p* < 0.05.

To control for multiplicity, we corrected for a 5% false discovery rate (FDR) across all comparisons via the Benjamini‒Hochberg procedure, whereby FRD-confirmed statistical significances appear in the results presentation. Effect size (ES) was measured by Hedges’ *g* due to different sample sizes, with effects classified as trivial (<0.2), small (≥0.2), moderate (≥0.6), large (≥1.2) and very large (≥2.0). The statistical power of the observations was calculated post-hoc with *α* = 0.05 when significant differences occurred and was found to be in the range 90%–100%. For non-significant comparisons, the observed power ranged 4%–60%, indicating the risk of type II errors in some position-specific analyses with very small sample sizes (e.g., GK *n* = 2–3). The means ± SD are reported, and 95% confidence interval (CI) of the mean differences are reported for key comparisons.

## Results

### Player characteristics

First, team players were naturally older (ES 2.60-very large, *p* < 0.001), but also taller (ES 0.58-small, *p* = 0.031) and heavier (ES 1.11-moderate) versus academy under-18 (*p* = 0.008) and reserve players (*p* = 0.040) (Table 1). Subgroup analysis showed this effect was particularly pronounced in AWs and FBs, whereas other player positions showed similar, but insignificant trends. In contrast, subcutaneous fat and adiposity did not differ between teams, including after sub-division into player positions, which suggests that the higher body mass in first-team players was due to higher lean mass.

IFT performance did not differ between teams, indicating comparable aerobic and anaerobic endurance and change of direction capacities, but the calculated V̇O_2max_ was ~7% (ES 1.09-moderate, *p* = 0.009 and *p* = 0.007 versus under-18 and reserve, respectively) higher in first-team players and ~10% (ES 1.55-large, *p* = 0.008 and *p* = 0.010 versus under-18 and reserve, respectively) higher after allometric scaling. Subgroup analysis showed this effect was especially pronounced in FBs, but a tendency for this effect was also observed in other player positions.

### Physical performance: Training

#### Distance

In a regular training session, under-18 players covered 727 m, 95% CI [345–1109] (ES 0.47-small, F(2,314) = 5.20, *p* = 0.006) more total distance than first-team players, whereas reserve players showed a similar, but insignificant trend (Table 2). This effect was also observed within some player positions.

Of the total distance, 2%–6% of this distance was in high-speed or sprint zones across the teams and player positions. Within these zones, under-18 players covered more high-speed distance than first-team players (ES 0.73-moderate, F(2,314) = 4.78, *p* = 0.009) and reserve players (ES 0.58-small, F(2,314) = 5.05, *p* = 0.007), as well as more sprint distance than first-team players (ES 0.84-moderate, F(2,314) = 12.24, *p* < 0.001) and reserve players (ES 0.57-small, F(2,314) = 9.64, *p* < 0.001), whereas no differences occurred between reserve and first-team players. This effect was also observed within some player positions.

#### Speed

Under-18 players achieved 0.63 m/s [0.34–0.92] (ES 0.54-small, F(2,314) = 4.88, *p* = 0.009) higher peak speeds than first-team players but not reserve players, whereas reserve players also tended to reach higher peak speeds than first-team players, but this difference did not reach statistical significance (Table 2). However, within specific player positions, this finding was less consistent.

We also calculated the mean speed of training sessions as an estimate of relative effort. For this, the situation was opposite to the above, as first team players achieved 8 m/min [4–12] (ES 0.52-small, F(2,314) = 3.79, *p* = 0.024) and 18 m/min [13–23] (ES 1.10–moderate, F(2,314) = 4.19, *p* = 0.016) higher values than under-18 and reserve players, respectively. Some player position variability was observed, but reserve players in most positions achieved the lowest mean speeds.

#### HR

No significant differences were observed between teams regarding time spent >85% of HR_max_, but subgroup analysis indicated that some player positions in under-18 and reserve teams to under-perform (Table 2).

### Physical performance: Match

#### Distance

In matches, academy players covered 5%–6% more distance than first-team players: under-18 539 m [56–1022] (ES 0.50-small, F(2,130) = 3.51, *p* = 0.034) and reserve 567 m [122–1012] (ES 0.53-small, F(2,130) = 3.49, *p* = 0.034) versus first-team players (Table 3). Among the total distance, 6%–10% of this was high-speed or sprint across the teams, with both under-18 (ES 0.71-moderate, F(2,130) = 4.88, *p* = 0.009) and reserve (ES 0.77-moderate, F(2,130) = 4.96, *p* = 0.008) players covering 18%–20% more high-speed distance than first-team players, but not more sprint distance. These effects were not attributed to specific player positions.

#### Speed

In line with the sprint distance, no peak speed differences were observed. For the mean speed, the situation differed from that of training, as both academy teams played matches with higher mean speeds than the first team: under-18 ES 1.11-moderate (F(2,130) = 3.33, *p* = 0.039) and reserve ES 1.20-large (F(2,130) = 4.04, *p* = 0.020) (Table 3). This was attributed to FB, CF and partly CB.

### Physical performance: Training versus match

**Figure 2.**
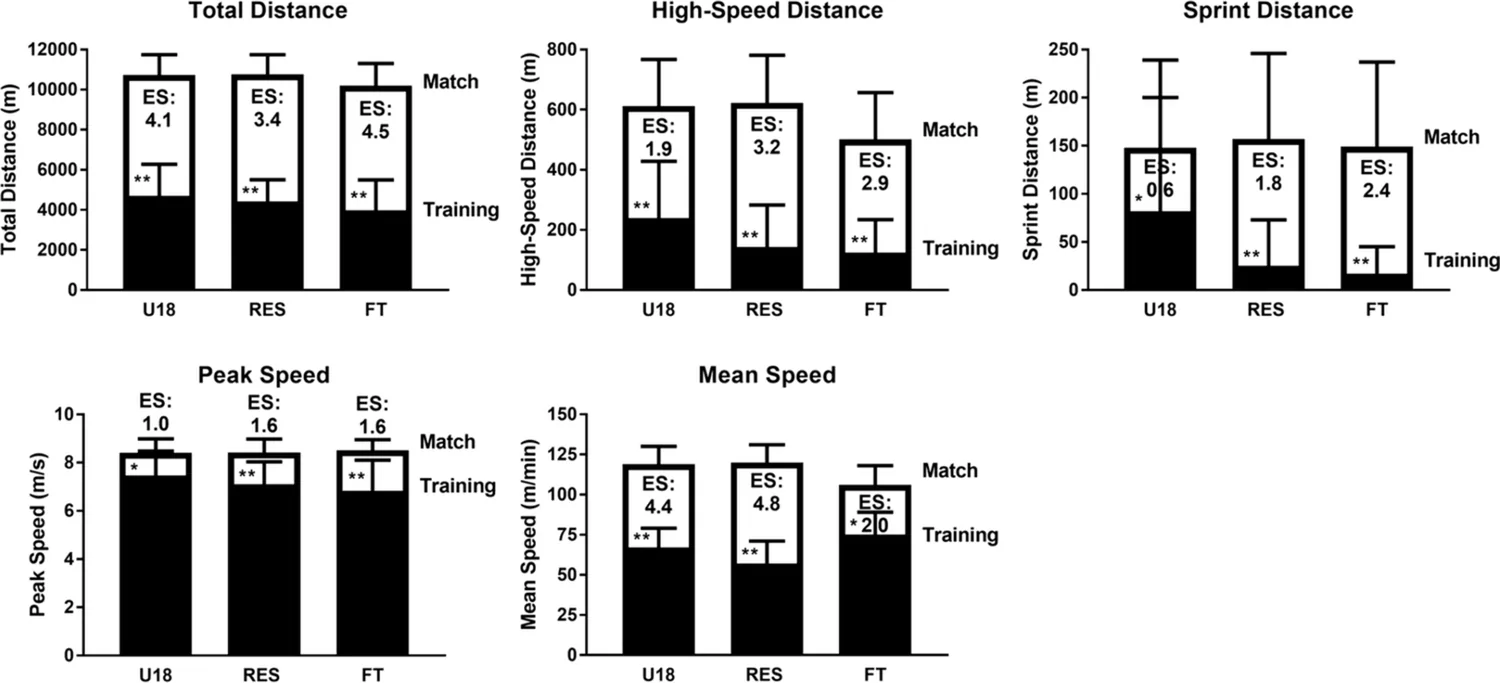
Physical performance in match versus training following a comparison of training data in Table 2 (black bars) versus match data in Table 3 (white bars). Abbreviations: U18 = under-18 team, RES = reserve team, FT = first team, ES = effect size. Statistical notes: Data are presented as the means ± standard deviation (SD). Statistical difference match versus training * = *p* < 0.05, ** = *p* < 0.01. The effect size (ES) of the difference between match and training for the individual team is numerically indicated on the bars.

All teams covered >100% (all *p* < 0.001) more distances in matches than in training (Figure 2: comparison of data in Tables 2 and 3), but training and match durations were not equalised, so this may not solely reflect effort. However, all teams also achieved >15% higher peak speeds and >40% higher mean speeds in matches than in training, though the ES of training-matched differences indicate that academy and especially under-18 players were closer to reaching match requirements in training versus first-team players in terms of high-intensity efforts (high-speed and sprint distances and peak speed), but first-team players were closer to reaching match requirements for relative effort (mean speed). Subgroup analyses showed that team findings were replicated across individual player positions.

### Age-dependent effects

Since the variances within each team are not evenly distributed (e.g., the first team has a wider range in terms of player age, experience and history of training and competitions versus academy teams), we extracted sub-category data from defined age-groups within the different teams, with cut-points informed by maturational transitions (Table 4). Albeit a trend commensurate with maturation was observed in youth players, no statistically significant differences occurred between the youngest and oldest under-18 team players. However, when the comparison between the oldest (≥18 years) reserve players versus the youngest (≤23 years) first-team players, an age-dependent effect occurred. The oldest reserve players covered more total (ES 0.92-moderate, *p* = 0.008) and high-speed distances (ES 1.13-moderate, *p* = 0.010) and achieved higher (ES 1.45-large, *p* = 0.004) mean speeds versus the youngest first-team players.

**Table 4.** Age-specific physical performance during a match in elite academy under-18 (U18) and reserve (RES) teams and first team (FT).

Number of players	U18		RES versus FT	
	≤16 yo *(16)*	>16 yo *(10)*	≥18 yo RES *(23)*	≤23 yo FT *(16)*
Total distance (m)	10129 ± 715	10702 ± 866	11287 ± 1037**	10248 ± 1253
95% CI	[−72, 1218]		[293, 1784]	
High-speed distance (5.5–7m/s, m)	599 ± 175	673 ± 174	642 ± 142**	489 ± 126
95% CI	[−72, 219]		[63, 243]	
Sprint distance (>7 m/s, m)	133 ± 58	164 ± 104	164 ± 107	154 ± 80
95% CI	[−34, 96]		[−54, 74]	
Peak speed (m/s)	8.21 ± 0.34	8.24 ± 0.41	8.61 ± 0.71	8.51 ± 0.50
95% CI	[-0.28, 0.34]		[−0.32, 0.52]	
Mean speed (m/min)	113 ± 8	119 ± 10	125 ± 12**	107 ± 13
95% CI	[−1, 13]		[10, 26]	

Notes: The table shows the physical performance outputs for the external load during the match in players from the academy U18 team segregated into younger and older age groups, and the oldest reserve team versus the youngest first-team players. Abbreviations: U18 = under-18 team, RES = reserve team, FT = first team, yo = years old, CI = confidence interval of the mean difference. Statistical notes: Data are presented as the means ± standard deviation. Statistical difference between cohorts (RES versus FT) after Benjamini‒Hochberg correction: ** = p<0.01.

### Current versus former academy players

Finally, we investigated whether current academy players are on a trajectory to reach the first team standard, compared to previous academy-first team transitioners at comparable stages of development. For this purpose, we identified 4 previous academy players who successfully transitioned to the first team and for whom we could retrieve comparable data (Table 5). This showed that current under-18 players in most parameters were comparable to former under-18 players, but also that current under-18 players had deficits in aerobic capacity (21% lower V̇O_2max_, ES 2.81-very large, *p* = 0.031) and relative effort exerted in training (109% lower mean speed, ES 6.15-very large, *p* = 0.016), whereas at the reserve team level, no differences occurred between current and former successful players.

**Table 5.** Former elite academy players that transitioned to first team (*n* = 4) versus current academy players (U18 *n* = 23, RES *n* = 15).

	Former academy – First team transition players			
	Former U18 Mean ± SD	*Δ* versus current U18 95% CI	Former RES Mean ± SD	*Δ* versus current RES 95% CI
**Player characteristics**				
Height (cm)	168.7 ± 8.8	−9.4 [−34.8, 16.0]]	184.2 ± 3.0	2.8 [−42.6, 48.2]
Body mass (kg)	59.2 ± 7.3	−9.8 [−33.8, 14.2]	72.2 ± 3.0	−2.3 [−50.4, 45.8]
Skinfolds (mm)	50.3 ± 0.6	−5.3 [−184.0, 173.4]	46.4 ± 4.5	−10.5 [−92.8, 71.8]
V̇O_2max_ (mL/kg/min)	63.9 ± 4.7 *	10.9 [4.5, 17.3]	56.8 ± 5.1	5.0 [−1.4, 11.4]
**Training performance**				
Total distance (m)	3716 ± 1879	−985 [−2143, 173]	5035 ± 1231	620 [−2262, 3502]
High-speed distance (5.5–7 m/s, m)	238 ± 302	−1 [−154, 152]	338 ± 206	195 [−783, 1173]
Sprint distance (>7 m/s, m)	44 ± 62	−38 [−134, 58]	137 ± 166	112 [−341, 565]
Peak speed (m/s)	6.75 ± 1.06	−0.72 [−1.50, 0.00]	7.98 ± 0.79	0.88 [−7.70, 9.40]
Mean speed (m/min)	140 ± 5 *	73 [62, 83]	88 ± 21	31 [−67, 129]
**Match performance**				
Total distance (m)	10721 ± 909	−13 [−2243, 2217]	10768 ± 717	6 [−12770, 12782]
High-speed distance (5.5–7 m/s, m)	545 ± 278	−67 [−369, 235]	519 ± 179	−103 [−2587, 2381]
Sprint distance (>7 m/s, m)	84 ± 67	−64 [−294, 166]	149 ± 119	−8 [−1565, 1549]
Peak speed (m/s)	7.60 ± 0.24	−0.81 [−3.70, 2.00]	8.36 ± 0.54	−0.06 [−8.50, 8.40]
Mean speed (m/min)	114 ± 13	−5 [−27, 17]	119 ± 14	−1 [−175, 173]

Notes: The table shows physical characteristics and physical performance outputs for external load during training and match in players from current academy U18 and RES teams versus academy-stage matched former players that subsequently successfully progressed to the first team. Abbreviations: U18 = under-18 team, RES = Reserve team, CI = confidence interval of the mean difference, V̇O_2max_ = maximal oxygen uptake. Statistical notes: Data are presented as the means ± standard deviation (SD). Statistical difference between cohorts (former versus current) after Benjamini‒Hochberg correction: * = *p*<0.05 versus current players.

## Discussion

Several systemic factors explain low academy-to-first team transition in professional top-level football (Carpels et al., 2021; Dugdale et al., 2021). However, the notion here was that factors related to physical fitness and performances in training and matching may also contribute and therefore to suggest an approach whereby individual academy players are assessed for physical readiness for academy-to-first team transfer in an objective and evidence-based manner. For this purpose, we compared physical fitness and performance profiles in training and matches between players of the respective teams, including after sub-division to player positions and age-groups and after comparison to previous successful academy-to-first team-transititioners. This generated benchmark requirements for individual players to meet in order to achieve upward transfer. These may be specific to the current club and context, but the approach will be transferable to other environments.

We found that academy under-18 and reserve players have not yet reached first-team level physical fitness and stature, but are largely able to produce comparable physical performance outputs in training and matches. We also identified performance behaviours that misaligned academy to first-team players, which may raise the possibility that academy players can be sub-optimally prepared for first-team transition. As such, this approach identifies specific deficits that may render current academy players less likely to achieve first team transition, but that may be overcome with appropriate training and conditioning. These development needs may vary between clubs and environments, but physical profiling will identify those and create local benchmarks that may inform and leverage local practice.

### Physical profiling

Previous studies have described similar benchmarking methods (Cone et al., 2012; Sporis et al., 2009) and examined physical characteristics and position-specific match demands (Bangsbo et al., 2006; Barnes et al., 2014; Bloomfield et al., 2007; Rampinini et al., 2007). However, these studies have not been stratified into different development stages or across academy-reserve-first team environments. We show for the first time that academy players have not yet developed comparable physical capacity to fully compete for the first team transition, nor have they fully developed body and lean mass, though the latter is not surprising (Milsom et al., 2015; Veale et al., 2010). Despite this, they show comparable physical performance in both training and match, although some performance behaviours differ between teams, e.g., high-intensity and average effort parameters. Such behaviour differences may, however, also be linked to context and different tactical objectives. Nonetheless, capacity differences between the academy and first teams may warrant rectification in academy players intending to progress to the first team.

### Physical performance in training and match

First, it adds external validity that training and match performances in the present study were comparable to European elite football reported previously, though below the very high-speed and sprint distances observed in the English Premier League (Barnes et al., 2014; Di Salvo et al., 2007; Lago-Penas et al., 2009; Rampinini et al., 2007).

Academy players performing at comparable or higher physical levels in training and matches than first-team players could be explained by several factors. Fixture frequencies and congestions vary, with the first team typically experiencing 2-matches/week and the academy teams 1-match/week microcycles, resulting in different load profiles (Carpels et al., 2024; Martin-Garcia et al., 2018; Swallow et al., 2021). Although we identified a 2-month period in the season with comparable fixture frequencies between teams for the match performance analysis, the different microcycles that dominate the season will likely also have affected training performance and accrued to spill-over affect match performances. As a result of the different microcycles across the season, the first team trained fewer sessions, but to compensate, they trained at higher mean speed and relative effort (normalised to duration) and thus higher intensity, in line with previous reports (Carpels et al., 2024; Houtmeyers et al., 2021). This ultimately suggests that scheduling constraints may also dictate between-team differences, which, in first team players, results in a higher level of alertness and engagement with training, a trait which academy players may still need to fully develop.

Match physical performance may also be influenced by variable contextual and opposition effects (Rampinini et al., 2007). This means that academy players tend to exhibit end-to-end action with more high-intensity movements throughout the full match because they prioritise individual education and development objectives, and they have less match management experience. In contrast, first team players tend to be governed by the collective objective of securing a good result while minimising physical effort (Kolodziejczyk et al., 2023). This was largely prevalent in this study, as the first team during the study period dominated and won the majority of matches and ended #2 in the domestic league. Although the academy teams also won the majority of their matches, match states and further contextual factors such as coaching priorities and tactics may ultimately still have contrasted player loads and match physical performances between teams.

### Subgroups

The observed differences in physical profiles and performances appeared as systematic trends within teams that were not explained by distinct player positions, albeit some position-specific differences were noted. In contrast, an expected maturation effect occurred across the academy, where older players demonstrated higher physical performances than their younger counterparts. Interestingly, though, the oldest reserve players also outperformed age-matched first team players, presumably because they were competing for individual first team transitions, whereas the youngest first team players already had achieved this. Overall, the subgroup analyses, and especially the comparison between current and former retro-profiled successful academy players, indicated that the current players were close to their trajectory to reach benchmark standards, but also highlighted areas for continued development. However, the above may reflect the composition of the current teams during this season. As such, indicators such as these may identify interesting player prospects rather than systemic biases, although the latter possibility should not be excluded if trends continue over several cohorts of players.

### Limitations

Interpretation of the current findings should be considered in light of key limitations, mainly single-club generalizability, unequal training and match contexts between teams, and analytical constraints.

We report a single-club study, a single-season study. As such, the specific quantitative findings should only be generalised to other clubs, leagues or developmental systems with caution, but the approach of applying comparative physical profiling to identify trends and development needs is generalizable. Additionally, subgroup analyses involved relatively small sample sizes due to player availability, which is inherently controlled by club operations and therefore difficult to control. While the primary conclusions derive from team-level data with adequate statistical power, subgroup results should be interpreted as indicative rather than definitive. Nevertheless, the broader approach of comparative physical profiling to identify developmental trends is transferable across performance environments.

Important contextual differences between teams with respect to training and matches limit comparisons. Different microcycles between first and academy teams (Carpels et al., 2024) means that between-team comparisons should be interpreted with caution. Practitioners should therefore incorporate contextual information when evaluating outcomes and making decisions. Furthermore, training sessions included exercises that may mask positional specificity, meaning that observed physical outputs may partly reflect individual behaviours rather than true position-specific demands. Also, training sessions were neither designed to fully replicate match conditions nor normalised to match durations. Consequently, the observation that training outputs do not match levels should not be interpreted as evidence of insufficient training *per se* but rather as a reflection of different contextual constraints. This discrepancy is particularly relevant in academic settings, where reduced match loads create the capacity for increased training loads.

Several analytical constraints also introduce additional uncertainty. Firstly, the use of historical data for retrospectively profiling previous academy players means that an unavoidable inter-device variability between systems (OptimEye X4 versus FieldWiz versus PlayerTek) may introduce systematic bias. While GPS technologies at these sampling frequencies have demonstrated acceptable validity (Johnston et al., 2014), we acknowledge that this comparison provides indicative benchmarks and contextual insight rather than absolute equivalence. Secondly, match data for players completing >60– <90 mins were normalised to the full match duration without adjustment for fatigue-related substitution effects, as substitutions may also have occurred due to tactical or injury-related factors (Rampinini et al., 2007). Thirdly, physical fitness was assessed using the 30:15 IFT. It provides an indirect estimate of cardiorespiratory fitness and V̇O_2max_, but is widely accepted in team sports for this purpose (Buchheit et al., 2011). Although test‒retest reliability was not established in this cohort, others have reported high reliability in comparable cohorts (see Materials and Methods for details). Finally, the study focuses exclusively on physical performance metrics; however, match outcomes and ultimately player development are also influenced by technical, tactical, and psychological factors (Morgans et al., 2023), but these factors are beyond the scope of this study.

### Practical applications

The described analytic approach can inform both short- and long-term decision-making within a club, helping it to identify players or cohorts who may require targeted support during their development pathway. This includes players who may benefit from adjustments in areas such as body composition, aerobic capacity and training or match performance behaviours. Importantly, the first team data presented here should not be interpreted as universal performance thresholds, but rather as club-specific reference values that can inform individualized development decisions within this particular performance environment.

These reference values are inherently shaped by contextual factors, including playing style, coaching philosophy, and training structure. For example, clubs employing counter-attack or high-press strategies may demonstrate different physical profiles compared to more position-oriented clubs. Accordingly, practitioners are encouraged to interpret and develop such reference values within their own club context, using them as a guide rather than as fixed standards. When applied appropriately, club-specific profiling may identify interesting academy prospects as well as areas of concern, enabling more informed and targeted follow-up interventions.

Although academy and first-team players in the present study demonstrated broadly comparable physical performance profiles in both training and match, it would be incorrect to state that academy players have no development requirements. Rather, the findings highlight the importance of individualised follow-up and development pathways, as performance gaps and strengths may vary between players. In practical terms, transitions from academy to first-team environments may be facilitated through progressive exposure to first-team requirements, allowing players time to adapt to higher or differently structured loads before full integration.

The observation that academy players can achieve comparable or, in some cases, higher training and match external performance outputs than first-team players despite lower physical capacities further underscores the need for careful monitoring of internal load and recovery statuses. This suggests that practitioners should consider individualised recovery strategies and load management approaches across teams to support fatigue resistance, performance in high-intensity match actions, and injury risk (Andrade et al., 2020; Martins et al., 2021). Finally, where academy players compare favourably to first-team players, communicating this achievement may have positive motivational benefits that boost confidence.

## Conclusions

Analytical physical profiling provides practical reference values from the first team that may be used to guide academy player development and assess and support transition readiness from a physical fitness and performance perspective. To optimise training and match practices, coaching staff should use these to target specific gaps in academy players while maintaining those aspects that already meet first-team standards. This ultimately allows for data-driven decision-making in clubs with regard to player logistics. Since these reference values are club- and context-specific, it is essential that each club establishes its own bespoke profiling frameworks.

## Data Availability

Data associated with this paper are available from the corresponding author upon reasonable request.

## References

[cit0001] Andrade, R. , Wik, E. H. , Rebelo-Marques, A. , Blanch, P. , Whiteley, R. , Espregueira-Mendes, J. , & Gabbett, T. J. (2020). Is the acute: Chronic workload ratio (ACWR) associated with risk of time-loss injury in professional team sports? A systematic review of methodology, variables and injury risk in practical situations. *Sports Medicine (Auckland, N.Z.)* , *50* (9), 1613–1635. 10.1007/s40279-020-01308-6 32572824

[cit0002] Apor, P. (1988). Successful formulae for fitness training. In T. Reilly , A. Lees , K. Davids , & W. J. Murphy (Eds.), *Science and Football* (pp. 95–107). London: E & FN Spon.

[cit0003] Bangsbo, J. , Mohr, M. , & Krustrup, P. (2006). Physical and metabolic demands of training and match-play in the elite football player. *Journal of Sports Sciences* , *24* (7), 665–674. 10.1080/02640410500482529 16766496

[cit0004] Barnes, C. , Archer, D. T. , Hogg, B. , Bush, M. , & Bradley, P. S. (2014). The evolution of physical and technical performance parameters in the English premier league. *International Journal of Sports Medicine (Stuttgart)* , *35* (13), 1095–1100. 10.1055/s-0034-1375695 25009969

[cit0005] Bloomfield, J. , Polman, R. , & O'Donoghue, P. (2007). Physical demands of different positions in FA premier league soccer. *Journal of Sports Science & Medicine* , *6* (1), 63–70. 24149226 PMC3778701

[cit0006] Bradley, P. S. , Sheldon, W. , Wooster, B. , Olsen, P. , Boanas, P. , & Krustrup, P. (2009). High-intensity running in English FA premier league soccer matches. *Journal of Sports Sciences* , *27* (2), 159–168. 10.1080/02640410802512775 19153866

[cit0007] Buchheit, M. (2008). The 30-15 intermittent fitness test: Accuracy for individualizing interval training of young intermittent sport players. *Journal of strength and conditioning research / National Strength & Conditioning Association* , *22* (2), 365–374. 10.1519/JSC.0b013e3181635b2e 18550949

[cit0008] Buchheit, M. , Lefebvre, B. , Laursen, P. B. , & Ahmaidi, S. (2011). Reliability, usefulness, and validity of the 30-15 intermittent ice test in young elite ice hockey players. *Journal of strength and conditioning research / National Strength & Conditioning Association* , *25* (5), 1457–1464. 10.1519/JSC.0b013e3181d686b7 21522077

[cit0009] Burgess, D. , Naughton, G. , & Norton, K. (2012). Quantifying the gap between under 18 and senior AFL football: 2003-2009. International Journal of Sports Physiology and Performance, *7* (1), 53–58. 10.1123/ijspp.7.1.53 21998145

[cit0010] Bush, M. , Barnes, C. , Archer, D. T. , Hogg, B. , & Bradley, P. S. (2015). Evolution of match performance parameters for various playing positions in the English premier league. *Human Movement Science* , *39* , 1–11. 10.1016/j.humov.2014.10.003 25461429

[cit0011] Carpels, T. , Scobie, N. , Macfarlane, N. G. , & Kemi, O. J. (2021). Youth-to-senior transition in elite european club soccer. *Int J Exerc Sci* , *14* (6), 1192–1203. 10.70252/JTGN8490 35096247 PMC8758176

[cit0012] Carpels, T. , Scobie, N. , Macfarlane, N. G. , & Kemi, O. J. (2024). Mind the gap: Comparison of external load and load variation between a reserve team in a 1-game week microcycle and its first team in a 2-game week microcycle within an elite professional soccer club. *Journal of strength and conditioning research / National Strength & Conditioning Association* , *38* (5), e235–e242. 10.1519/JSC.0000000000004734 38517476

[cit0013] Clemente, F. M. , Silva, R. , Arslan, E. , Aquino, R. , Castillo, D. , & Mendes, B. (2021). The effects of congested fixture periods on distance-based workload indices: A full-season study in professional soccer players. *Biology of Sport* , *38* (1), 37–44. 10.5114/biolsport.2020.97068

[cit0014] Cone, J. R. (2012). Soccer-specific performance testing of fitness and athleticism: The development of a comprehensive player profile. *Strength and Conditioning Journal* , *34* (5), 11–19. 10.1519/SSC.0b013e3182575e8c

[cit0015] Dalen, T. , & Loras, H. (2019). Monitoring training and match physical load in junior soccer players: Starters versus substitutes. *Sports (Basel)* , *7* (3), 70. 10.3390/sports7030070 30893911 PMC6473774

[cit0016] Dellal, A. , Lago-Penas, C. , Rey, E. , Chamari, K. , & Orhant, E. (2015). The effects of a congested fixture period on physical performance, technical activity and injury rate during matches in a professional soccer team. *British Journal of Sports Medicine (Guildford)* , *49* (6), 390–394. 10.1136/bjsports-2012-091290 23422422

[cit0017] Di Salvo, V. , Baron, R. , Tschan, H. , Calderon Montero, F. , Bachl, N. , & Pigozzi, F. (2007). Performance characteristics according to playing position in elite soccer. *International Journal of Sports Medicine (Stuttgart)* , *28* (3), 222–227. 10.1055/s-2006-924294 17024626

[cit0018] Dugdale, J. H. , Sanders, D. , Myers, T. , Williams, A. M. , & Hunter, A. M. (2021). Progression from youth to professional soccer: A longitudinal study of successful and unsuccessful academy graduates. *Scandinavian Journal of Medicine and Science in Sports* , *31* (Suppl 1), 73–84. 10.1111/sms.13701 33871087

[cit0019] Gilgen-Ammann, R. , Schweizer, T. , & Wyss, T. (2019). RR interval signal quality of a heart rate monitor and an ECG holter at rest and during exercise. *European Journal of Applied Physiology* , *119* (7), 1525–1532. 10.1007/s00421-019-04142-5 31004219

[cit0020] Grgic, J. , Lazinica, B. , & Pedisic, Z. (2021). Test-retest reliability of the 30-15 intermittent fitness test: A systematic review. *The Journal of Sport and Health Science* , *10* (4), 413–418. 10.1016/j.jshs.2020.04.010 32422345 PMC8343059

[cit0021] Helgerud, J. , Engen, L. C. , Wisloff, U. , & Hoff, J. (2001). Aerobic endurance training improves soccer performance. *Medicine and Science in Sports and Exercise (Baltimore, MD)* , *33* (11), 1925–1931. 10.1097/00005768-200111000-00019 11689745

[cit0022] Houtmeyers, K. C. , Jaspers, A. , Brink, M. S. , Vanrenterghem, J. , Varley, M. C. , & Helsen, W. F. (2021). External load differences between elite youth and professional football players: Ready for take-off?. *Science and Medicine in Football* , *5* (1), 1–5. 10.1080/24733938.2020.1789201 35073232

[cit0023] Johnston, R. J. , Watsford, M. L. , Kelly, S. J. , Pine, M. J. , & Spurrs, R. W. (2014). Validity and interunit reliability of 10 hz and 15 hz GPS units for assessing athlete movement demands. *Journal of strength and conditioning research / National Strength & Conditioning Association* , *28* (6), 1649–1655. 10.1519/JSC.0000000000000323 24276300

[cit0024] Kolodziejczyk, M. , Chmura, P. , Modric, T. , Kołodziejczyk, M. , Versic, S. , Andrzejewski, M. , Sekulic, D. , Rokita, A. , & Konefał, M. (2023). Do players competing in the UEFA champions league maintain running performance until the end of the match? Positional analysis between halfes and 5-minute intervals. *Journal of Sports Medicine and Physical Fitness* , *63* (3), 394–401. 10.23736/S0022-4707.22.14069-7 35816145

[cit0025] Koudellis, M. , Tsouloupas, C. , Christou, M. , Aphamis, G. , Bogdanis, G. C. , & Giannaki, C. D. (2024). Physical fitness, psychological characteristics, and game performance in youth Male soccer players of different levels of competition. *International Journal of Performance Analysis in Sport* , *25* , 290–304. 10.1080/24748668.2024.2411107

[cit0026] Lago-Penas, C. , Rey, E. , Lago-Ballesteros, J. , Casais, L. , & Dominguez, E. (2009). Analysis of work-rate in soccer according to playing positions. *International Journal of Performance Analysis in Sport* , *9* (2), 218–227. 10.1080/24748668.2009.11868478

[cit0027] Malone, J. J. , Di Michele, R. , Morgans, R. , Burgess, D. , Morton, J. P. , & Drust, B. (2015). Seasonal training-load quantification in elite English premier league soccer players. *International Journal of Sports Physiology and Performance* , *10* (4), 489–497. 10.1123/ijspp.2014-0352 25393111

[cit0028] Martin-Garcia, A. , Gomez Diaz, A. , Bradley, P. S. , Morera, F. , & Casamichana, D. (2018). Quantification of a professional football team's external load using a microcycle structure. *Journal of strength and conditioning research / National Strength & Conditioning Association* , *32* (12), 3511–3518. 10.1519/JSC.0000000000002816 30199452

[cit0029] Martins, A. D. , Oliveira, R. , Brito, J. P. , Loureiro, N. , Querido, S. M. , & Nobari, H. (2021). Intra-season variations in workload parameters in Europe's elite young soccer players: A comparative pilot study between starters and non-starters. *Healthcare (Basel)* , *9* (8), 977. 10.3390/healthcare9080977 34442114 PMC8392320

[cit0030] Meckel, Y. , Doron, O. , Eliakim, E. , & Eliakim, A. (2018). Seasonal variations in physical fitness and performance indices of elite soccer players. *Sports (Basel)* , *6* (1), 14. 10.3390/sports6010014 29910318 PMC5969193

[cit0031] Milsom, J. , Naughton, R. , O'Boyle, A. , O’Boyle, A. , Iqbal, Z. , Morgans, R. , Drust, B. , & Morton, J. P. (2015). Body composition assessment of English premier league soccer players: A comparative DXA analysis of first team, U21 and U18 squads. *Journal of Sports Sciences* , *33* (17), 1799–1806. 10.1080/02640414.2015.1012101 25686107

[cit0032] Morgans, R. , Orme, P. , & Di Michele, R. (2023). Impact of technical and physical performance on match outcome over five elite european soccer seasons. *Journal of Sports Medicine and Physical Fitness* , *63* (3), 417–429. 10.23736/S0022-4707.22.14018-1 35816143

[cit0033] Nassis, G. P. , Massey, A. , Jacobsen, P. , Brito, J. , Randers, M. B. , Castagna, C. , Mohr, M. , & Krustrup, P. (2020). Elite football of 2030 will not be the same as that of 2020: Preparing players, coaches, and support staff for the evolution. *Scandinavian Journal of Medicine and Science in Sports* , *30* (6), 962–964. 10.1111/sms.13681 32424904

[cit0034] Rampinini, E. , Coutts, A. J. , Castagna, C. , Sassi, R. , & Impellizzeri, F. M. (2007). Variation in top level soccer match performance. *International Journal of Sports Medicine (Stuttgart)* , *28* (12), 1018–1024. 10.1055/s-2007-965158 17497575

[cit0035] Reilly, T. , George, K. , Marfell-Jones, M. , Scott, M. , Sutton, L. , & Wallace, J. (2009). How well do skinfold equations predict percent body fat in elite soccer players?. *International Journal of Sports Medicine (Stuttgart)* , *30* (8), 607–613. 10.1055/s-0029-1202353 19301213

[cit0036] Sporis, G. , Jukic, I. , Ostojic, S. M. , & Milanovic, D. (2009). Fitness profiling in soccer: Physical and physiologic characteristics of elite players. *Journal of strength and conditioning research / National Strength & Conditioning Association* , *23* (7), 1947–1953. 10.1519/JSC.0b013e3181b3e141 19704378

[cit0037] Swallow, W. E. , Skidmore, N. , Page, R. M. , & Malone, J. J. (2021). An examination of in-season external training load in semi-professional soccer players: Considerations of one and two match weekly microcycles. *The International Journal of Sports Science & Coaching* , *16* (1), 192–199. 10.1177/1747954120951762

[cit0038] Theodoropoulos, J. S. , Bettle, J. , & Kosy, J. D. (2020). The use of GPS and inertial devices for player monitoring in team sports: A review of current and future applications. *Orthopedic Reviews* , *12* (1), 7863. 10.4081/or.2020.7863 32391130 PMC7206363

[cit0039] Tounsi, M. , Wiewlhove, T. , Racil, G. , Gervasi, M. , Frizziero, A. , Migliaccio, G. M. , Padulo, J. , & Trabelsi, Y. (2024). Reference values of specific physical performances of elite youth Male soccer players: Taking into consideration the maturity status. *Muscles, Ligaments and Tendons Journal* , *14* (3), 417–429. 10.32098/mltj.03.2024.05

[cit0040] Ulijaszek, S. G. , & Kerr, D. A. (1999). Anthropometric measurement error and the assessment of nutritional status. *British Journal of Nutrition (London)* , *82* (3), 165–177. 10.1017/s0007114599001348 10655963

[cit0041] Veale, J. P. , Pearce, A. J. , Buttifant, D. , & Carlson, J. S. (2010). Anthropometric profiling of elite junior and senior Australian football players. *International Journal of Sports Physiology and Performance* , *5* (4), 509–520. 10.1123/ijspp.5.4.509 21266735

[cit0042] Waldron, M. , & Murphy, A. (2013). A comparison of physical abilities and match performance characteristics among elite and subelite under-14 soccer players. *Pediatric exercise science* , *25* (3), 423–434. 10.1123/pes.25.3.423 23877584

